# Retraction Note: Efficacy and safety of water-free topical cyclosporine for moderate to severe dry eye disease: a systematic review and meta-analysis

**DOI:** 10.1186/s12348-025-00556-9

**Published:** 2025-11-13

**Authors:** Ahmed Hamed Rehan, Hassan El-Masry, Roaa Abdultawab, Manahil Ahmed, Rawan Ashraf Kasem, Mohammad Al Diab Al Azzawi, Shree Rath

**Affiliations:** 1 Kasr Alainy Faculty of Medicine, Cairo University, Cairo, Egypt; 2 Faculty of Medicine, Alexandria University, Alexandria, Egypt; 3 Faculty of Medicine, Ain Shams University, Cairo, Egypt; 4 Jinnah Sindh Medical University (JSMU), Karachi, Pakistan; 5 Faculty of Medicine, The National Ribat University, Khartoum, Sudan; 6 All India Institute of Medical Sciences, Bhubaneswar, Odisha India; 7 Research Web Platform (ResWeb), Cairo, Egypt

**Retraction Note: Journal of Ophthalmic Inflammation and Infection (2025) 15:20**

10.1186/s12348-025-00467-9

The Editor in Chief has retracted this article following concerns about calculation errors in Figs. 4, 5 and 6. The authors have acknowledged the errors and are invited to resubmit a new version of the manuscript that does not contain these errors. The submission will be subjected to peer review. Rawan Ashraf Kasem, Hassan El-Masrym Shree Rath, Mohammad Al Diab Al Azzawi, Roaa Abdultawab and Manahil Ahmed agree to this retraction. Ahmed Hamed Rehan has not responded to correspondence about this retraction.

